# Cholesterol regulation of mechanosensitive ion channels

**DOI:** 10.3389/fcell.2024.1352259

**Published:** 2024-01-25

**Authors:** Katie M. Beverley, Irena Levitan

**Affiliations:** Division of Pulmonary, Critical Care, Sleep, and Allergy, Department of Medicine, College of Medicine, University of Illinois at Chicago, Chicago, IL, United States

**Keywords:** cholesterol, mechanosensitive ion channels, Piezo, Kir2.1, TRPV4

## Abstract

The purpose of this review is to evaluate the role of cholesterol in regulating mechanosensitive ion channels. Ion channels discussed in this review are sensitive to two types of mechanical signals, fluid shear stress and/or membrane stretch. Cholesterol regulates the channels primarily in two ways: 1) indirectly through localizing the channels into cholesterol-rich membrane domains where they interact with accessory proteins and/or 2) direct binding of cholesterol to the channel at specified putative binding sites. Cholesterol may also regulate channel function via changes of the biophysical properties of the membrane bilayer. Changes in cholesterol affect both mechanosensitivity and basal channel function. We focus on four mechanosensitive ion channels in this review Piezo, Kir2, TRPV4, and VRAC channels. Piezo channels were shown to be regulated by auxiliary proteins that enhance channel function in high cholesterol domains. The direct binding mechanism was shown in Kir2.1 and TRPV4 where cholesterol inhibits channel function. Finally, cholesterol regulation of VRAC was attributed to changes in the physical properties of lipid bilayer. Additional studies should be performed to determine the physiological implications of these sterol effects in complex cellular environments.

## 1 Introduction

Cholesterol is a major lipid component of plasma membranes in all mammalian cells and has a well-established role of regulating ion channel function (Yeagle, 1985; Levitan et al., 2014; Zakany et al., 2020; Jiang and Levitan, 2022). Several general mechanisms have been shown to underlie cholesterol regulation of ion channels (Levitan et al., 2014; Zakany et al., 2020; Jiang and Levitan, 2022): (i) direct binding of the cholesterol molecule to non-annular hydrophobic pockets of the channel (Rosenhouse-Dantsker et al., 2013; Barbera et al., 2018); (ii) indirectly by altering the biophysical properties of the membranes, such as membrane fluidity and stiffness (Lundbaek et al., 1996; Romanenko et al., 2004a) or (iii) enhancing protein-protein interactions that are proposed to occur within the environment of cholesterol-rich membrane domains, termed lipid rafts (O’Connell et al., 2004). However, it is also important to note that these domains are still controversial because the nature of these domains remains unclear including size, morphology, stability, and composition (Anderson and Jacobson, 2002). These important controversies, specifically the difficulty in accurately isolating these domains, and potential resolutions for the field are also discussed by Levental et al., 2020. There is strong evidence though that cholesterol may regulate proteins including ion channels via facilitating protein-protein interactions, which is consistent with the idea of cholesterol rich scaffolding domains (Sheng et al., 2012; Qi et al., 2015).

In the majority of cases, an increase in membrane cholesterol suppresses the activity of the channels, which was demonstrated for a variety of ion channels, including inwardly rectifying K^+^ channels, Kir2 (Romanenko et al., 2004b; Epshtein et al., 2009), Kir3.1 (Bukiya and Rosenhouse-Dantsker, 2017) and Kir6.2 (Csonka et al., 2014), transient receptor potential vanilloid cation channels, TRPV1 (Picazo-Juarez et al., 2011), TRPV4 (Morin et al., 2022), large conductance Ca2^+^-sensitive K^+^ channels (Bukiya et al., 2011). However, some channels, specifically several members of transient receptor potential (Trpc) channels family (Trpc1 and Trpc3) (Brownlow and Sage, 2005), Piezo channels (Ridone et al., 2020) and some Kir channels (Kir3.4 and Kir7.1) (Rosenhouse-Dantsker et al., 2010) are inhibited by removing cholesterol from the membrane indicating that the presence of cholesterol is required for the channel function. Cholesterol suppression of the channels was shown to be mediated primarily by the direct binding of cholesterol to the channel proteins and in some cases by an increase in the stiffness of the lipid bilayer, whereas inhibition of the channel activities by cholesterol removal is attributed primarily to the disruption of the protein-protein interactions within cholesterol-rich domains.

Here, we discuss the effects of cholesterol on mechanosensitive ion channels, those that are activated by mechanical forces, such as shear stress, a frictional force generated by the blood flow and by membrane tension generated by stretch. As one of the first responders to mechanical forces, mechanosensitive ion channels are strong candidates to constitute primary mechanosensors that transduce mechanical signals to initiating cell signaling cascades leading to physiological responses (Gerhold and Schwartz, 2016; Kefauver et al., 2020). In the last decade, Piezo1 channels have been identified as a major type of mechanosensitive channels, activated directly by the mechanical signals and sensitive to both shear stress and stretch (Ranade et al., 2014; Lhomme et al., 2019). Multiple studies also showed that Piezo1 channels play a major role in endothelial mechanotransduction (Ranade et al., 2014). Another type of mechanosensitive ion channels that is getting increased attention is Kir2.1, which has been long known to be sensitive to shear stress (Olesen et al., 1988) and more recently identified as a key modulator of endothelial response to flow (Ahn et al., 2017; Fancher et al., 2018; Fancher et al., 2020; Ahn et al., 2022). TRPV4 channels have also been shown to be sensitive to flow in mouse endothelial cells where they transduce shear stress into calcium signaling leading to flow-mediated dilation of small resistance arteries (Mendoza et al., 2010). Additionally, endothelial volume regulated ion channels (VRAC) are regulated by flow (Barakat et al., 1999; Alghanem et al., 2021) but their impact on endothelial mechanotransduction is not well understood yet. Multiple studies evaluated the role of membrane cholesterol on the function of these channels, but a comprehensive review of these studies is still lacking. In this article, we discuss direct and indirect effects of cholesterol on the ion channel structure and function of mechanosensitive ion channels, based on computational data sets and physiological findings. We also discuss the physiological relevance of cholesterol effects on ion channels and highlight new research directions and gaps in current knowledge.

## 2 Cholesterol regulates piezo channels by altering its microenvironment

PIEZO channels are mechanosensitive cation channels which carry calcium, potassium, and sodium and are activated by membrane stretch, compression and shear stress (Liu et al., 2022). It is the canonical stretch-activated ion channel and a primary regulator of mechanotransduction. Piezo1 is a trimeric protein with a pore in the middle. The membrane lipids surrounding the protein generate a dome-like structure and uses the trimeric structure of the Piezo1 channel as a lever to mediate mechanical force. Inflammatory conditions have been shown to induce mechanical stress which is sensed by Piezo1 (Liu et al., 2022). In endothelial cells, Piezo1 is necessary for development of blood vessels by activating Akt and Erk signaling facilitating cell alignment and stretch mediated calcium influx (Wang et al., 2021).

**FIGURE 1:**
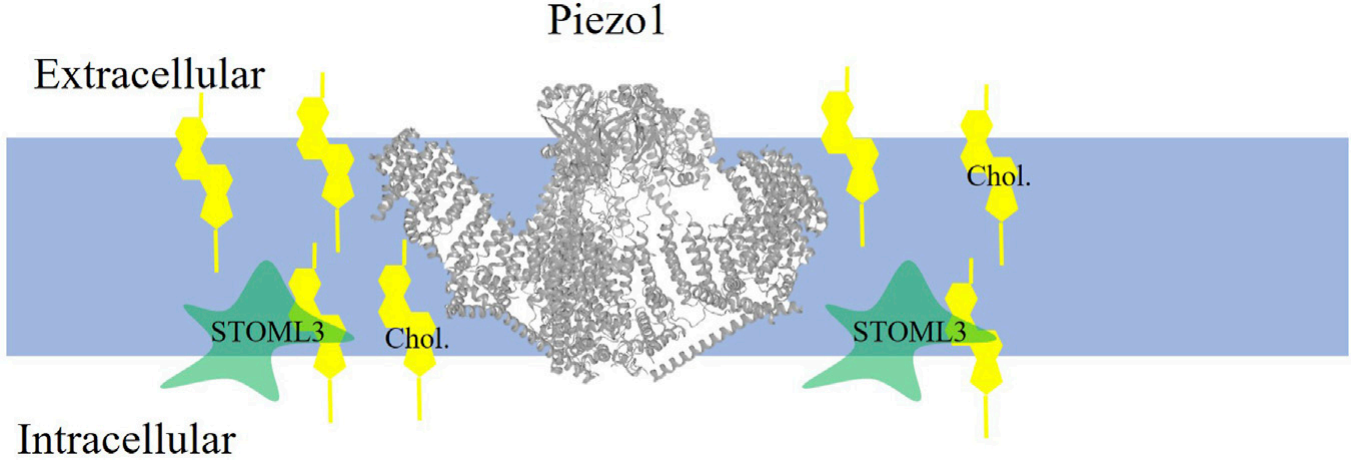
Piezo1 is localized to cholesterol-rich domains where it is indirectly enhanced by cholesterol. Piezo1 channel (PDB:5z10 rendered in PROTEAN3D) function is enhanced when cholesterol (yellow symbol) binds to the accessory protein STOML3.

Sensitivity of Piezo1 stretch-activated mechanosensitive channels to cholesterol was demonstrated by showing that depletion of membrane cholesterol using methyl-β-cyclodextrin (MβCD), a cyclic oligosaccharide with high affinity to cholesterol, results in a marked suppression of the stretch-activated currents in mouse sensory neurons (Qi et al., 2015). The mechanism of this effect was identified to be critically dependent on an axillary protein, stomatin-like protein-3 (STOML3) (schematically shown in Figure 1), which was shown previously to be essential for touch-induced mechanotransduction and to regulate piezo channels (Qi et al., 2015). Qi et al. (2015) showed that STOML3 is associated with low-density high cholesterol membrane fractions, generally termed lipid rafts, and that STOML3 deficiency and depletion of cholesterol result in a similar reduction in the stretch-activated currents with no additive effect suggesting that cholesterol and STOML3 regulate the channels via the same pathway. This hypothesis was tested by generating a STOML3 mutant (P40S) deficient in the ability to bind cholesterol, which was found to disrupt the localization of STOML3 in the low-density membrane fractions and abolish its effect on endogenous stretch-activated currents in the neurons. Furthermore, a similar effect was observed in N2a neuroblastoma cells that express very low endogenous level of STOML3: overexpressing WT STOML3 strongly potentiated stretch-activated currents, whereas cholesterol-binding deficient mutant of STOML3 did not. These observations indicate that STOML3 binding to cholesterol is essential for its regulation of stretch-activated currents in sensory neurons. To address more directly the role of cholesterol in regulation of piezo channels, piezo2 channels were co-expressed with WT or cholesterol-binding deficient STOML3 in heterologous expression system, HEK293 cells, leading to the same result: cholesterol depletion attenuated the potentiation of the currents by WT STOML3 but not by its cholesterol-binding deficient mutant. Thus, clearly, cholesterol plays a major role in the interaction of the channels with STOML3, which in turn plays a key role in the mechanosensitivity of the channels. Conversely, the authors conclude that cholesterol regulates mechanosensitivity of piezo channels via its interaction with STOML3: when cholesterol is depleted, it disrupts the integrity of cholesterol-rich membrane domains/lipid rafts leading STOML3 to redistribute in the membrane and that disrupts piezo-STOML3 interaction. Furthermore, the authors propose that STOML3 regulate mechanosensitity of the channels via regulating biomechanical properties of the plasma membrane. This conclusion is based on their findings that downregulation of STOML3, as well as cholesterol depletion, results in a decrease in the elastic modulus of the membrane, which might be detrimental to the ability of the channels to sense stretch. More specifically, the authors propose that STOML3 facilitates the recruitment of cholesterol to the plasma membrane, resulting in membrane stiffening, which in turn facilitates the transmission of tension. This is definitely an intriguing idea but more evidence is needed to support it.

More recently, the effect of cholesterol depletion on the biophysical properties and clustering of piezo1 channels was analyzed when the channels were heterologously expressed in HEK293 cells (Ridone et al., 2020) or endogenously expressed in mouse neuroblasoma cells (N2A cells) (Qi et al., 2015). They demonstrated that exposure to MβCD slows down the activation of the channels in response to stretch, resulting in decreased open probability of the channels and the loss of their mechanosensitivity. Specifically, when cholesterol is depleted, Piezo1 channels are not activated in response to negative pressure applied to a membrane patch, as shown in cell-attached patch clamp configuration in stably expressing Piezo1-Hek293 cells (Ridone et al., 2020). Additionally, treating the cells with the dynamin inhibitor dynasore, a compound known to disrupt cholesterol-rich membrane domains (Preta et al., 2015) is shown to have the same effect on piezo1 channels, as MβCD-induced cholesterol depletion (Ridone et al., 2020). In contrast, increasing membrane cholesterol using MβCD-complexed with cholesterol had no significant effect on piezo1 activity, which is consistent with the idea that the channels depend on the integrity of cholesterol-rich membrane domains. Ridone et al. (2020) also found that cholesterol depletion increases the diffusion rate of the channel proteins and decreases the clustering of the channels, visualized by STORM, as well as the loss of the co-localization of the channels with GM1, a known marker of cholesterol-rich domains/lipid rafts. These data led the authors to propose that cholesterol depletion breaks the local cholesterol scaffold and removed the channels from their local environment. Interestingly, some of the effects of cholesterol depletion of the biophysical properties of piezo1 channels were found to be similar to a known mutation of the channels (R2456H), which is associated with hereditary xerocytosis (Bae et al., 2013). In addition, in Piezo1 knockout mice there is a decrease in expression of genes encoding enzymes associated with cholesterol oxidation and esterification including Cyp46a1, Ch25h, and Soat2 (Nourse et al., 2022). This finding is suggestive of a positive feedback mechanism where continued expression and activation of the Piezo1 channel leads to increase free cholesterol within the membrane.

In addition, putative cholesterol binding sites have been identified on piezo2 channels by Molecular Dynamic simulations (Lin et al., 2022) suggesting that direct binding of cholesterol may also contribute to the regulation of the channels. These binding sites were found on the N-terminal domain and as hydrophobic pockets on the transmembrane domains and are distinct from the established cholesterol-binding domains that contain CRAC or CARC motifs (Fantini and Barrantes, 2013). Importantly, the functional consequences of this direct binding has not been determined.

Thus, in summary it was found that cholesterol depletion potentiates mechanosensitivity of piezo channels by regulating their microenvironment, although by different mechanisms that might be mutually dependent, complementary or distinct.

## 3 Cholesterol suppresses flow-sensitive Kir channels by direct binding to the channel protein

Early studies by Olesen et al. (1988) identified endothelial Kir channels to be activated by fluid shear stress within seconds after the application of flow leading to the hypothesis that these channels constitute primary shear stress sensors (Ahn et al., 2017; Fancher et al., 2020; Ahn et al., 2022). More recently, it was further established that Kir2 channels are essential for flow-induced release of nitric oxide, a major vasodilatory and anti-inflammatory agent and flow-induced vasodilation (Ahn et al., 2017; Ahn et al., 2022). Furthermore, impairment of endothelial Kir2 channels by hypercholesterolemia and obesity was found to be a major driver of endothelial dysfunction (Fancher et al., 2018; Fancher et al., 2020).

In contrast to piezo channels, flow-sensitive Kir2.1 channels are suppressed by cholesterol as shown by altering cholesterol content of the membrane using MβCD or MβCD-cholesterol complex to deplete or enrich cellular cholesterol respectively (Romanenko et al., 2002; Romanenko et al., 2004a). The suppression effect was demonstrated for all members of Kir2 family although to a different degree with Kir2.1 and Kir2.2 being most sensitive (Romanenko et al., 2004a). Furthermore, endothelial Kir channels were also shown to be suppressed by the pro-atherogenic very low density lipoproteins (VLDL) and modified acetylated low density lipoproteins (acLDL), both known to increase cellular cholesterol (Fang et al., 2006). More recently, we also found that exposing endothelial cells to elevated levels of low density lipoproteins (LDL), the major cholesterol carrier in the blood, results in loading endothelial cells with cholesterol and suppression of Kir currents (Ahn et al., 2022). Cholesterol-induced suppression of Kir currents was also observed in endothelial cells freshly-isolated from hypercholestreolemic animal models, diet-induced hypercholesterolemia in pigs (Fang et al., 2006) and genetically-induced hypercholesterolemia in ApoE^−/−^ mice (Fancher et al., 2018). In the porcine model, endothelial cells were isolated from aortas of hypercholesterolemic and control pigs and the electrophysiological experiments were performed immediately after the isolation on the day of the animal sacrifice. Kir currents in endothelial cells isolated from aortas of hypercholesterolemic pigs were significantly lower than those in cells isolated from aortas of control animals and the suppression was recovered by cholesterol depletion with MβCD indicating that the observed suppression of the currents was cholesterol dependent. Similarly, Kir currents were also significantly reduced in mesenteric endothelial cells freshly-isolated from ApoE^−/−^ mice, as compared to wild type controls (Fancher et al., 2018). Also, similarly to the earlier study in hypercholesyterolemic pigs, suppression of Kir current in endothelial cells isolated from ApoE^−/−^ mice was fully rescued by depleting cellular cholesterol with MβCD. Notably, Kir currents in endothelial cells isolated from ApoE^−/−^ mice also lost their sensitivity to flow (Ahn et al., 2022).

There are three lines of evidence that suggest cholesterol regulates Kir channels by direct binding: (i) distinct effects cholesterol versus its chiral analogues, epicholesterol and ent-cholesterol on the channel function, (ii) sensitivity of purified bacterial Kir channels to cholesterol in liposomes, and (iii) identification of specific cholesterol binding sites on Kir2 channel proteins. In early studies, it was shown that exposing endothelial cells to MβCD complexed with epicholesterol, which resulted in a partial (∼50%) substitution of endogenous cholesterol with its chiral analogue led to a significant increase in Kir current density, that was even higher than in cholesterol-depleted cells (Romanenko et al., 2002). Since cholesterol and epicholesterol have similar effects on the biophysical properties of the membrane lipid bilayer, the enhancement of Kir current was interpreted as competition between the chiral analogues for binding either to the Kir channel protein directly or to an auxiliary protein regulating channel function (Romanenko et al., 2002). Similarly, the partial substitution of cholesterol with its other chiral analogue, ent-cholesterol, also showed that Kir channels are sensitive to the chiral nature of cholesterol (D’Avanzo et al., 2011). Furthermore, reconstituting purified bacterial Kir channels, KirBac1.1, into artificial liposomes with a defined lipid composition allowed to test whether cholesterol regulation of the channels depends on any auxiliary proteins or signaling pathways present in a complex environment of the cellular milieu (Singh et al., 2009). Channel function was evaluated by measuring 86Rb + uptake into liposomes. This study showed that cholesterol suppresses KirBac 1.1 in the absence of any accessory proteins (Singh et al., 2009). In a follow-up study, direct binding to KirBac1.1 was shown with radioactive cholesterol. The main findings were that the binding is saturable, that non-radioactive cholesterol competes with radioactive cholesterol for binding to the channel, that cholesterol binding to KirBac1.1 is reversed by the same pharmacological inhibitor that blocks cholesterol binding to SCAP1 and that inhibition of cholesterol binding led to the rescue of KirBac1.1 activity, clearly indicating that cholesterol-induced suppression of KirBac1.1 function is mediated by cholesterol binding to the channel protein (Singh et al., 2011).

**FIGURE 2:**
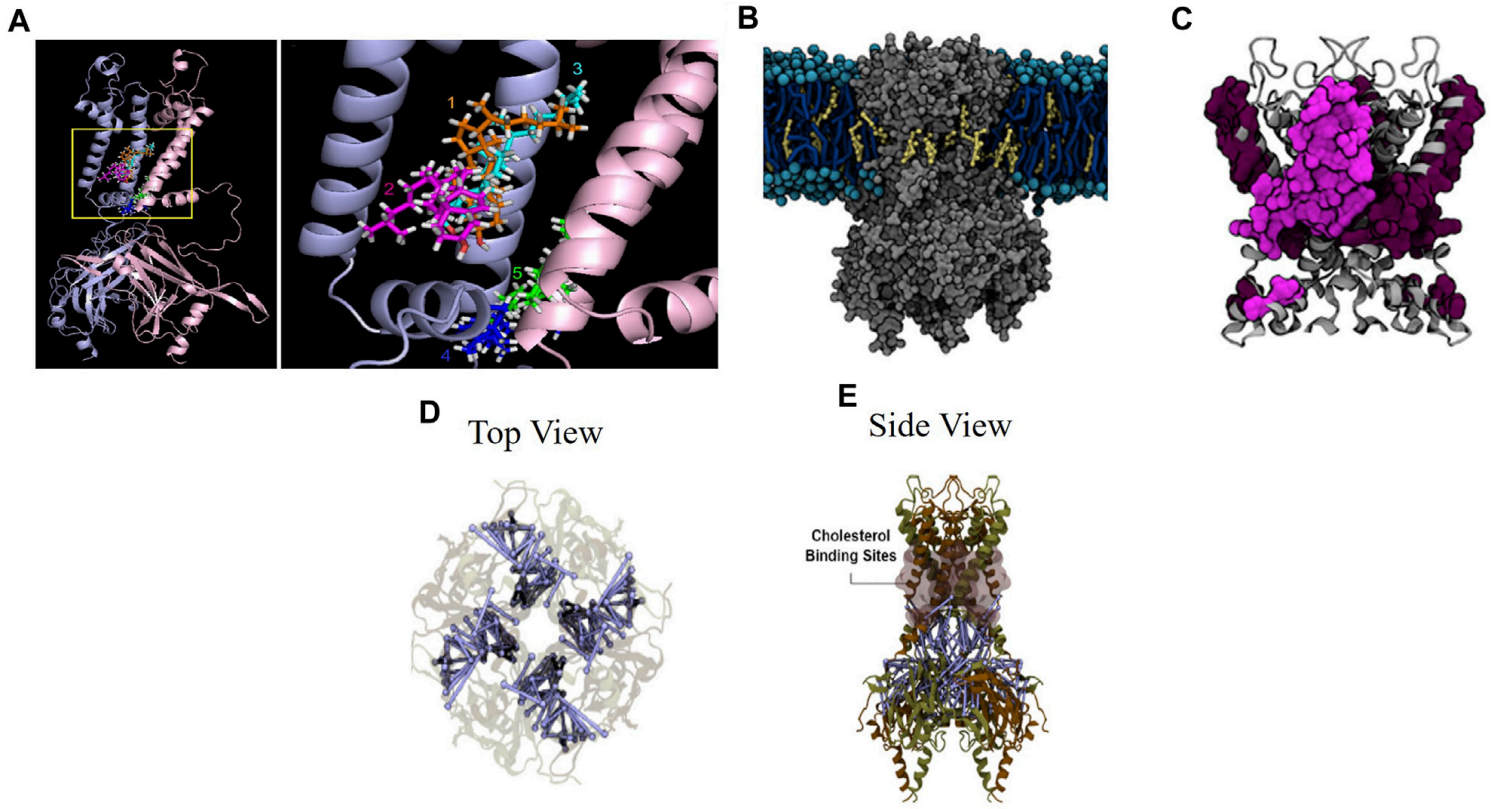
Cholesterol suppresses Kir2.2 through direct binding to the channel. **(A)** Five cholesterol binding clusters from all-atom MD solutions in the transmembrane and transmembrane-cytosolic interface in the Kir2.1 channel chimera (Rosenhouse-Dantsker et al., 2013). **(B)** Cholesterol interacting with Kir2.1 in the cell membrane (Barbera et al., 2018). **(C)** Zoomed view of cholesterol binding residues in the transmembrane and transmembrane-cytosol regions. Residues from a single subunit are highlighted (Barbera et al., 2018). **(D)** (top view) and **(E)** (side view) of close-state Kir2.2 with blue lines indicating residue uncoupling, shaded pink surfaces indicating previously identified cholesterol binding sites, and brown and tan indicate individual subunits (Barbera et al., 2022).

Finally, putative cholesterol binding sites have been identified using molecular dynamics simulations. In our earlier study (Rosenhouse-Dantsker et al., 2013), two putative cholesterol binding regions were identified in Kir2.1 channel using a homology model based on the chimeric structure of the cytosolic domain of Kir3.1 and the transmembrane domain of KirBac1.3, partial structures of Kir channels available at that time (Nishida et al., 2007; Tai et al., 2009). Since cholesterol is expected to interact with the proteins within the lipid membrane, molecular docking studies focused in the transmembrane domains of the channel protein and the domains on the membrane-cytosolic interface. The docking analysis resulted in numerous poses of the cholesterol molecule interacting with the channel, which can be expected because a small lipid molecule, such as cholesterol, might have multiple hydrophobic interactions with the channel protein. Further analysis showed that these poses formed 6 clusters (4 between the transmembrane domains and 2 on the interface between the transmembrane helices and the slide helix of the N-terminus on the membrane-cytosol interface. However, since molecular docking has significant limitations, specifically, they do not allow the channel protein to adjust its conformation when the cholesterol molecules bind, the results of the docking analysis were fine-tuned using all-atom molecular dynamics (MD) simulations. Indeed, during the MD simulations, the clusters coalesced into two cholesterol binding regions: clusters 1–3 coalesced into region 1 in the transmembrane domain and clusters 4–5 coalesced into region 2 on the membrane-cytosolic interface (Figure 2A). The 6th cluster appeared to be a false positive. Notably, the simulations showed that cholesterol continuously changes its pose interacting with the channel protein in a highly dynamic way. The predictions from the simulations were supported by site-directed mutagenesis and electrophysiology demonstrating that a number of residues in both regions (L85, V93, S95, I166, V167, I175, and M183 in region 1 and L69, A70, and V77 in region 2) are critical for the sensitivity of the channels to cholesterol. Importantly, these sites are not part of the structured belt of cholesterol-sensitive residues around the cytoplasmic pore. Additionally, the binding sites identified in this study are outside of the established CARC and CRAC cholesterol binding motifs (Rosenhouse-Dantsker et al., 2013).

We followed these studies up with longer course-grained MD simulations (Barbera et al., 2018) for which we used the open and close states of the Kir2.2 structure (Tao et al., 2009; Hansen et al., 2011). These simulations unlike the previous study evaluated the channel protein in the context of the cell membrane and allowed the cholesterol molecules to diffuse freely within the membrane and interact with the channel in a dynamic way (without pre-set docking). We observed that cholesterol interacted with Kir2.2 channels in short-term interactions on the surface of the channel and in long-term interactions in the hydrophobic pockets of the channel. There were also multiple cholesterol molecules that were bound to the channel at any given time. Importantly, using this unbiased approach, we identified cholesterol binding sites similar to those reported in our earlier study that relied on pre-docked cholesterol, which shows consistency and robustness of the analysis. In this study, we also found that the binding sites in the open and in the closed conformation of Kir2.2 partially overlap with multiple common residues between the two conformation states (L83, L84, S87, L88, A89, L91, V163, V164, C170, I172, and F175) but are also distinct with the residues that were unique to the open state (V92, S93, I167, V168, I171) and conversely unique to the closed state (M70, F71, C74, M82, L85, F86, F90, M176, I180). These cholesterol binding sites are shown in Figures 2B, C. Reduced membrane cholesterol resulted in a decrease in the quantity of cholesterol molecules interacting with the channel and a decrease in the interaction time at the identified cholesterol binding residues (Barbera et al., 2018). The findings from these structural studies were supported by multiple site-directed mutations which were evaluated by whole-cell patch clamp electrophysiology. Taken together, these studies provide evidence indicating that Kir2.1 and Kir2.2 are suppressed by cholesterol through direct binding to the channel protein independent of accessory proteins.

To evaluate functional consequences of cholesterol binding to Kir2.2 channels, additional computational analysis was performed to evaluate structural changes in the channel as a result of cholesterol binding (Barbera et al., 2022). The findings showed a decrease in overall contact probability between residues at the inter-subunit interface in a high cholesterol condition (Barbera et al., 2022), which have been identified in earlier studies to be important for Kir2 gating (Pegan et al., 2005; Clarke et al., 2010; Gupta et al., 2010; Lacin et al., 2017). Specifically, the residue contact probability decreases with an increase in number of cholesterol molecules bound to the channel. The uncoupled residues are shown relative to the cholesterol binding sites in Figures 2C–E. Point mutations in specific uncoupled residues where subunits interact resulted in abrogation or reversal of cholesterol suppression of Kir2.2 channel current as shown by high throughput electrophysiology (Barbera et al., 2022). This study provides a structure-based mechanism for the cholesterol induced Kir2.2 channel suppression based on residue uncoupling at inter-subunit interfaces.

In terms of the physiological importance of cholesterol-induced suppression of Kir2.1, we showed that hypercholesteremia in Apoe^−/−^ mice led to the loss of Kir2.1-dependent NO production and flow-induced vasodilation in mesenteric arteries (Fancher et al., 2018). These effects were reversed by cholesterol depletion or overexpression of Kir2.1 in mouse endothelial cells (Fancher et al., 2018). Even stronger evidence came from generating a whole-body CRISPR mouse model where endogenous wild type Kir2.1 was substituted with its mutant insensitive to cholesterol due to a point mutation where leucine is changed to isoleucine at position 222 (L222I) (Ahn et al., 2022). This mutation was identified in the previous structure-function studies that identified the C-terminal domain as important for cholesterol sensitivity, specifically the CD loop (Epshtein et al., 2009). Ahn et al. (2022) showed that the cholesterol insensitive L222I mutation is protective of flow-induced vasodilation in mesenteric arteries of ApoE^−/−^ mice and rescues Kir2.1 channel current and mechanosensitivity in hypercholesteremic conditions in mouse endothelial cells. Taken together these studies highlight the physiological importance of Kir2.1 cholesterol sensitivity in cardiovascular disease.

## 4 TRPV4 are regulated by direct cholesterol binding and by cholesterol binding-dependent partition into caveolae

TRPV4 channels are mechanosensitive non-selective cation channels, sensitive to flow and membrane tension (Rosenbaum et al., 2020). In addition, TRPV4 channels are activated by cell swelling in response to a hypo-osmotic stimulus, an effect that was attributed to membrane stretch (Rosenbaum et al., 2020). The TRPV4 channel regulates intracellular calcium homeostasis, which plays important roles in the endothelial barrier maintenance, regulation of vascular tone, and osmoregulation, as well as nocioception (Rosenbaum et al., 2020). TRPV4 also play critical roles in bone homeostasis, pulmonary, and kidney function (Rosenbaum et al., 2020). TRPV4 channels have been demonstrated to be flow-sensitive through two mechanisms; direct activation by membrane tension by hypotonic swelling (Liedtke et al., 2000; Lakk et al., 2021) and an indirect effect of channel translocation to the cell membrane induced by a release of calcium from the intracellular stores (Baratchi et al., 2016).

It has been shown that TRPV4 is inhibited by an increase in cellular cholesterol in multiple cell types, including endothelial cells, bone cells and *xenopus* oocytes, whereas cholesterol depletion resulted in increased current amplitude (Das and Goswami, 2019; Lakk et al., 2021; Morin et al., 2022). Cholesterol was also shown to suppress the activation of TRPV4 channels by both agonists and mechanical stimuli (shear stress) in mouse mesenteric arteries and in human coronary artery endothelial cells (Mendoza et al., 2010). Cholesterol depletion was also found to enhance the calcium influx response to a TRPV4 agonist GSK1016790A in Muller glial cells (Lakk et al., 2017).

**FIGURE 3:**
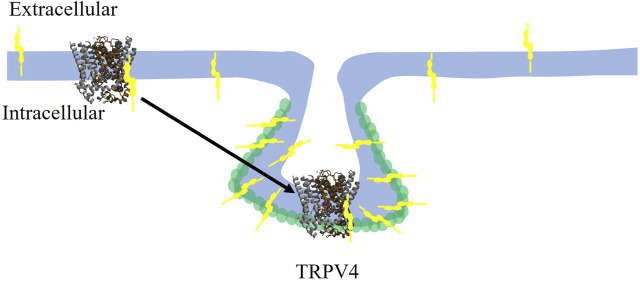
Cholesterol suppresses the TRPV4 channel by direct binding and trafficking the channel to caveolae. Cholesterol (yellow symbol) binds to TRPV4 in the membrane and traffics it to the caveolae where it colocalizes with Cav-1 (green circles). TRPV4 channel rendering adapted from TRPV1 in Barbera et al., 2019.

Similarly to cholesterol regulation of Kir2 channels, several lines of evidence support TRPV (TRPV1 and TRPV4) channels to be regulated by direct binding of cholesterol to the channel protein. First, earlier studies showed that cholesterol inhibits TRPV1 channel, a homologue of TRPV4 via direct binding (Picazo-Juarez et al., 2011). This was demonstrated by a combination of computational docking studies followed by site-directed mutagenesis and electrophysiological recordings. It was found that cholesterol molecule docks to a cholesterol-binding consensus CARC motif of TRPV1 and that mutations of specific residues of this domain led to the loss of cholesterol sensitivity of TRPV1 (Picazo-Juarez et al., 2011). Consistent with these findings, cholesterol was also found to dock to a conserved CARC motif located in the TM4-loop-TM5 domain of TRPV4 (Das and Goswami, 2019). In both channels, cholesterol may interact with its putative binding domains in multiple poses. A mutation in the TRPV4 CARC motif, R616Q renders the channel to become cholesterol insensitive, indicating that this site is critical for cholesterol-induced suppression of TRPV4 function. Furthermore, while TRPV4 wild type channel co-localizes with caveolin-1 indicating its partition into caveolae (see schematic depiction in Figure 3), TRPV4-R616Q mutant does not co-localize with caveolin-1 suggesting that cholesterol binding is required for its interaction with caveolin-1 and partitioning into caveolae. Interestingly, TRPV4 WT molecular weight was found to be higher than that of the TRPV4-R616Q mutant further suggesting that TRPV4 WT interacts with other proteins whereas TRPV4- R616Q loses these interactions, which is also consistent with TRPV4 WT partitioning into the caveolae, whereas TRPV4-R616Q is not. It is also important to note that R616Q mutation in TRPV4, has been associated with brachyolmia in humans, a rare bone disorder (Verma et al., 2010) suggesting that an over-active cholesterol insensitive TRPV4 plays a role in this disease (Das and Goswami, 2019).

The association of TRPV4 with caveolae may not be universal though: while as described above, TRPV4 was found to co-localize with caveolin-1 in bone and mesenchymal stem cells (Das and Goswami, 2019), it was found not to co-localize with caveolin-1 in tubercular meshwork epithelia cells and in *xenopus* oocytes (Lakk et al., 2021). However, in spite of not partitioning to caveolae, the channels are still sensitive to the level of cellular cholesterol with cholesterol enrichment suppressing and cholesterol depletion enhancing TRPV4 activity, indicating that partitioning into caveolae is not required for cholesterol sensitivity of the channels (Lakk et al., 2021).

Functionally, TRPV4-cholesterol interactions have been implicated in enhanced vasodilation in rat femoral arteries under hypoxic conditions (Morin et al., 2022). This is based on the observation that the contribution of TRPV4 to acetylcholine-induced vasodilation is enhanced under the hypoxic conditions, which was shown earlier to result in a decrease in membrane cholesterol in endothelial cells (Naik and Walker, 2018), whereas an increase in membrane cholesterol by exposing the arteries to MβCD-cholesterol, the contribution of TRPV4 to the vasodilation decreases (Morin et al., 2022). These studies provide functional evidence for the role of TRPV4 cholesterol sensitivity in vascular function.

## 5 Cholesterol indirectly suppresses VRAC

Volume Regulated Anion channels (VRAC) are well known to be activated by cell swelling, which suggests that they are activated by stretch in numerous cell types (Osei-Owusu et al., 2018). Specifically, VRAC channels become active with cell expansion in hypo-osmotic conditions, allowing Cl^−^ ions and negatively-charged organic osmolytes to efflux from the cell to the extracellular milieu. The efflux of these ions reduces the osmotic pressure within the cells allowing the cells to undergo regulatory volume decrease, which brings cell volume back to normal (Jaeger et al., 1999; Okada et al., 2006). In terms of the sensitivity of VRAC to cell swelling, it has been argued that the mechanism of channel activation could be either membrane stretch or a decrease in the ionic strength of the intracellular milieu or decrease in macromolecule crowding (Okada et al., 2006). Importantly, VRAC’s were also shown to be activated by fluid shear stress generated by flow in vascular endothelial cells, indicating that these channels are flow/mechanosensitive (Barakat et al., 1999; Barakat et al., 2006). In terms of the molecular identity of VRAC channel, it was a topic of extensive studies and multiple controversies but only recently it was definitively identified as hetero-hexamers comprised of the five members of the LRRC8 channel family (Pedersen et al., 2016; Osei-Owusu et al., 2018). LRRCA is a critical subunit for physiological function of VRAC’s and heteromerization with other subunits LRRC8B-E contributes to the biophysical properties of the channel (Syeda et al., 2016).

In terms of cholesterol sensitivity, similarly to Kir2 and TRPV channels, VRAC were shown to be inhibited by cholesterol enrichment and enhanced by cholesterol depletion (Levitan et al., 2000). In contrast to Kir2 channels, however, VRAC were insensitive to the chiral nature of cholesterol, as substitution of the endogenous cholesterol with its structural analogue, epicholesterol, showed no effect on the current. Further studies that replaced cholesterol with its structural analogues showed that VRAC activity correlates with changes in membrane biophysical properties leading to the conclusion that cholesterol-induced suppression of VRAC can be attributed to an indirect effect based on membrane deformation energy rather than by direct binding (Romanenko et al., 2004b).

## 6 Conclusion

Multiple types of mechanosensitive ion channels are regulated by membrane cholesterol via three general mechanisms: direct binding of the cholesterol molecule to the channel protein, regulation by the biophysical properties of the lipid bilayer and interaction with auxiliary proteins enhanced by partitioning of the channels into cholesterol-rich membrane domains. Notably, the direct binding mechanism that was demonstrated for Kir2 and TRPV4 channels, as well as regulation by cholesterol-mediated changes in the properties of the bilayer (VRAC) result in the inhibitory effect on the channel function, whereas regulation by the interaction with auxiliary proteins within the environment of cholesterol-rich membrane domains, as demonstrated by Piezo1, results in the enhanced activity of the channels. Importantly, as discussed in detail above, changes in membrane cholesterol not only regulate the basal activities of the channels but also have a major impact on their mechanosensitivity: enhanced mechanosensitivity of Piezo1 channels in response to stretch and suppressed mechanosensitivity of Kir2 in response to flow and of TRPV4 in response to stretch or cell swelling. Further studies are needed to elucidate the physiological impact of these effects in complex cellular environments when multiple types of mechanosensitive channels are expressed.
